# Impact of Screen Time on Development of Children

**DOI:** 10.3390/children12101297

**Published:** 2025-09-25

**Authors:** Subhranshu Sekhar Kar, Rajani Dube, Bellary Kuruba Manjunatha Goud, Qonitah Syadida Gibrata, Adlen Adnan El-Balbissi, Tasnimm Ahmad Al Salim, Rand Nedal Mohammad Al Khaled Fatayerji

**Affiliations:** 1 Department of Pediatrics, RAK Medical and Health Sciences University, Ras Al Khaimah 11172, United Arab Emirates; subhranshu.kar@rakmhsu.ac.ae (S.S.K.); adlenadnan77@gmail.com (A.A.E.-B.); tasneemsalim954@gmail.com (T.A.A.S.); ranood425@gmail.com (R.N.M.A.K.F.); 2 Department of Obstetrics and Gynecology, RAK Medical and Health Sciences University, Ras Al Khaimah 11172, United Arab Emirates; rajani.dube@rakmhsu.ac.ae; 3 Department of Biochemistry, RAKCOMS, RAK Medical and Health Sciences University, Ras Al Khaimah 11172, United Arab Emirates; manjunatha@rakmhsu.ac.ae

**Keywords:** screen time, child development, pediatric health, cognitive development, emotional development

## Abstract

Background: Children today are growing up in a digital environment where screen-based technology is a central part of everyday life. While screens can offer educational and recreational benefits, there is growing concern about their influence on different areas of child development. Objective: This review explored how screen time affects developmental outcomes in children and adolescents aged 0 to 18 years, focusing on physical, cognitive, emotional, and social domains. Methods: A structured search was carried out across databases such as PubMed, Scopus, Web of Science and PsycINFO to identify relevant studies published between 2014 and 2024. Studies were included if they examined the relationship between screen time and at least one area of child development, involved participants within the target age group, and were peer-reviewed and published in English. The review followed PRISMA guidelines, and articles were independently screened and evaluated for quality by two reviewers. Results: A total of 46 studies met the inclusion criteria. Overall, the evidence points to a link between higher levels of screen use and negative outcomes such as reduced physical activity, poorer sleep, attention difficulties, and challenges in emotional and social functioning. However, some studies indicated that limited or educational screen use, especially with parental involvement, may have neutral or even positive effects in certain contexts. Conclusions: Screen time can have both positive and negative effects on child development, depending on factors like duration, type of content, and the context in which screens are used. Managing screen use through age-appropriate guidelines and adult supervision may help reduce risks and promote healthier development. More longitudinal research is needed to establish clearer recommendations for screen use in childhood.

## 1. Introduction

Screen time has become a necessary component of daily living in the current digital age, especially for kids [1,2]. Since cell phones, tablets, laptops, and televisions are so common, kids are exposed to digital media at a young age [3]. Even though screen time has educational and entertainment value, worries about how technology affects kids’ development are becoming more and more prevalent. Concerns over the impact of excessive screen time on cognitive, social, emotional, and physical development are growing among experts and parents [2,3,4]. According to research, screen time can have both positive and negative effects, depending on the child’s age, the type of content viewed, and the length of exposure. Overuse of screens has been connected to issues like lack of physical exercise, attention problems, language delays, and impaired social skills [2,4]. However, some forms of screen time in the classroom can help students acquire abilities like digital literacy and problem-solving [4].

Current evidence on the effect of mobile phone radiation (radiofrequency electromagnetic fields, RF-EMF) and congenital problems is limited and inconclusive [5,6,7]. Unlike ionizing radiation (X-rays, gamma rays), RF-EMF is non-ionizing and does not have enough energy to directly damage DNA. Large cohort studies (e.g., Danish National Birth Cohort, MoBa study in Norway) have not shown strong associations between maternal mobile phone use and major congenital malformations [8,9,10,11]. Some studies suggested possible links to behavioral problems or subtle neurodevelopmental effects in children, but results are not consistent [8,9].

The purpose of this narrative review was to examine the body of evidence on the effects of screen usage on children’s development. It highlighted how screen time affects children’s cognitive, social, and emotional development and provided advice on how to appropriately manage screen time to support kids’ general well-being. This analysis looked at the complex relationship between screen time and child development in an effort to guide future studies and help parents, guardians, and legislators make wise choices regarding their kids’ screen time.

## 2. Materials and Methods

This review summarized studies examining how children’s screen usage affects their cognitive, emotional, social, and physical development. A range of study designs and data sources were examined in order to provide a comprehensive grasp of this subject.

### 2.1. Study Inclusion Criteria

The selection of studies was based on their emphasis on the association between screen time exposure and developmental outcomes, such as behavior, emotional control, physical health, and cognitive capacities, in children ages 0–18. To cover various research viewpoints, the review incorporated randomized controlled trials (RCTs), cross-sectional studies, and longitudinal studies.

### 2.2. Data Collection

PubMed, Scopus, Web of Science, PsycINFO and other databases were the sources of the examined papers. Most of the studies included were papers from medical and psychological societies, reports from health organizations, and peer-reviewed journals. The review included studies from the last 10 years.

### 2.3. Research Methodology

A variety of research designs were used:

- Quantitative research: These studies primarily used surveys and questionnaires to assess screen time exposure (length, kind of material, and devices used) to examine developmental outcomes like academic achievement, cognitive abilities, and behavioral changes.
- Qualitative research: To learn more about how parents, guardians, and educators perceive the effects of screen usage on kids, researchers interviewed these participants. Family dynamics and the emotional impacts of screen time were also examined through focus groups.
- Mixed-methods research: Some researchers blended quantitative and qualitative approaches, merging statistical analysis with in-depth interviews and observations to offer a more holistic perspective.

### 2.4. Measured Outcomes

The research evaluated a number of developmental outcomes, such as the following:

- Cognitive development: memory, language acquisition, and academic success.
- Emotional and social development: Social skills, emotional control, behavioral adaptations, and the growth of empathy.
- Physical health metrics: Body mass index (BMI), activity level, sleep quality, and the development of motor skills.
- Mental health: Problems such as anxiety, depression, and the psychological effects of excessive screen usage.

### 2.5. Data Analysis Techniques

The data was analyzed using a variety of statistical methods, e.g., descriptive statistics to compile the study populations’ characteristics and screen time exposure. Regression analysis was used to ascertain how screen time and developmental outcomes are related.

### 2.6. Criteria for Study Selection

#### Methodology

A comprehensive literature search was conducted to explore the impact of screen time on children’s development. The inclusion criteria targeted the following types of studies:

1. Involving children and adolescents aged 0 to 18 years;
2. Examining the relationship between screen use (e.g., screen time, digital media exposure) and developmental outcomes (physical, social, emotional, or cognitive);
3. Published in peer-reviewed journals within the last 10 years (1 January 2015–31 December 2024);
4. Written in English.

Databases searched included PubMed, Scopus, Web of Science and PsycINFO. Boolean operators were used to construct the search strategy. The search string used was as follows:

(“screen time” OR “screen use” OR “digital media” OR “media exposure” OR “electronic media”)

AND (“child*” OR “adolescent*” OR “infant*” OR “toddler*” OR “teen*” OR “youth”)

AND (“development” OR “cognitive development” OR “social development” OR “emotional development” OR “physical development”)

AND (“impact” OR “effect*” OR “association*” OR “relationship*”)

Filters were applied to limit the results to English-language articles published in the past 10 years in peer-reviewed journals. The study selection process involved screening titles and abstracts, followed by full-text review. Duplicates and irrelevant studies were removed.

**Figure 1:**
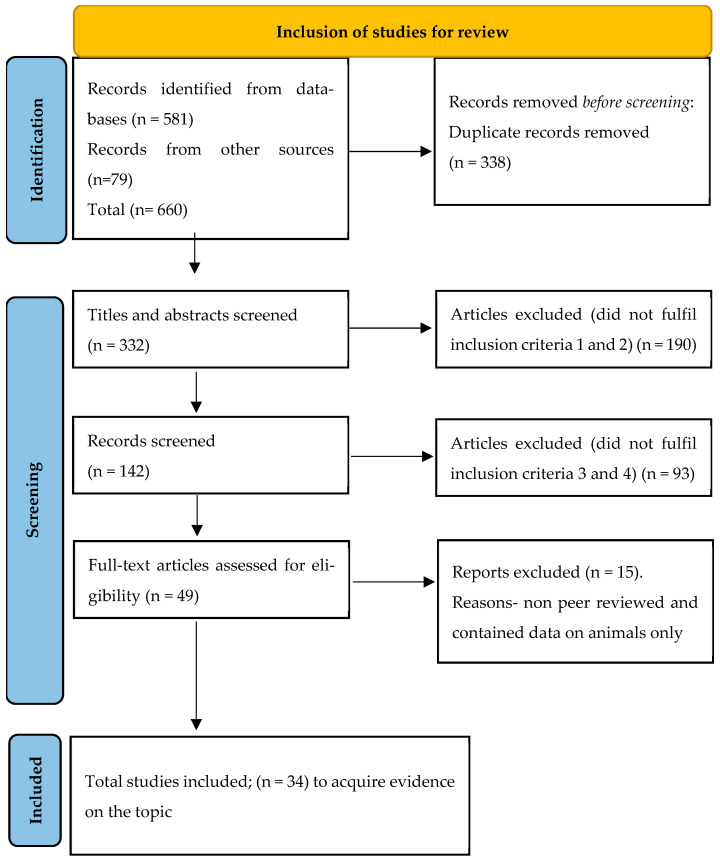
PRISMA flow diagram for study inclusions.

A PRISMA flow diagram (Figure 1) summarizes the selection process.

## 3. Results and Discussion

A total of thirty-four studies were included in Table 1, exploring the impact of screen time on development of children.

### 3.1. Understanding Screen Time

#### 3.1.1. Definition of Screen Time

“Screen time” refers to the amount of time individuals spend using devices with screens—such as smartphones, tablets, computers, televisions, and gaming consoles. Activities range from passive consumption (e.g., watching television, browsing social media) to interactive engagement (e.g., educational games, coding, or creative digital projects) [1].

Types of screens include the following:

- Smartphones and tablets: Portable devices used for communication, entertainment, and productivity.
- Computers and laptops: Common tools for work, education, and digital entertainment.
- Televisions: Initially used for broadcasting news and entertainment, now widely used for streaming digital content.
- Gaming consoles: Devices primarily designed for video gaming, offering both recreational and social experiences.

#### 3.1.2. Guidelines and Recommendations for Screen Time Usage by Age Group

To support parents and caregivers, the American Academy of Pediatrics (AAP) and other experts offer guidance tailored to children’s developmental stages (Table 2) [2,3].

**Figure 2:**
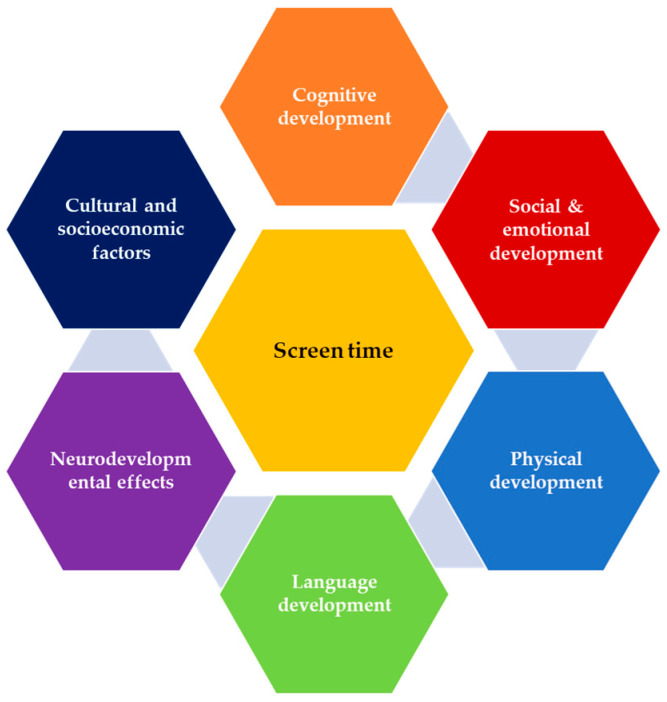
Multidimensional impact of screen time on child development.

Figure 2 represents the multidimensional impact of screen time on child development, highlighting several interconnected domains.

### 3.2. Cognitive Development

The increasing integration of digital devices into children’s routines raises important questions about their effect on cognitive development, defined as the set of mental processes that enable learning, reasoning, problem-solving, and understanding. Though digital media presents risks, it can also offer developmental benefits if used appropriately [4,44,45].

#### 3.2.1. Positive Aspects of Screen Time on Cognitive Development

Not all screen time is detrimental. When used mindfully, certain digital tools can promote creativity, critical thinking, and problem-solving. Educational programs such as “Sesame Street” have been shown to support early math and literacy skills [46]. Interactive apps that focus on storytelling, puzzles, or coding can enhance memory, cognitive flexibility, and attention span. Studies demonstrate that high-quality media exposure can have measurable cognitive benefits. For instance, children exposed to educational programming often exhibit stronger language development and better problem-solving skills than those who consume non-educational media [47].

#### 3.2.2. Negative Effects of Screen Time on Cognitive Development

Conversely, excessive or low-quality screen time may have adverse effects. Fast-paced or overstimulating content—like action-filled video games or rapid-fire cartoons—has been associated with reduced attention span and difficulty focusing on sustained tasks [19]. A particularly concerning area is language development. Children under two who engage in more passive media use tend to have delayed language acquisition, possibly due to less interaction with caregivers [48]. Excessive screen use has also been linked to deficits in executive function—including planning, self-regulation, and delayed gratification [49]. Activities that reward instant responses, such as games or scrolling feeds, may impair children’s ability to engage in thoughtful, goal-directed behavior.

#### 3.2.3. Content and Context of Screen Time

The quality and context of screen use are just as important as quantity. Interactive and educational content promotes engagement and learning, while passive viewing can be detrimental [50]. According to the AAP, limiting screen time to one hour per day for children aged 2–5, and ensuring consistent limits for older children, can help maintain developmental balance [2]. Parental involvement remains essential. When caregivers participate by co-viewing, discussing content, or playing educational games, screen time becomes a more socially and cognitively enriching experience. In contrast, solitary screen use is associated with delays in language acquisition and social skill development [51].

### 3.3. Social and Emotional Development

Social and emotional development refers to a child’s ability to understand and manage emotions, establish healthy relationships, and navigate social environments. This foundation is essential not only for emotional well-being but also for long-term success in school, relationships, and life. Table 3 summarizes the effect of screen time on social and emotional development in children [12,13,14,28,51,52,53,54,55,56,57,58,59,60].

### 3.4. Physical Development

Physical development is a foundational component of overall child health. It encompasses growth, motor skill acquisition, and the formation of long-term health behaviors. With the increasing dominance of screen time in children’s routines, there are growing concerns about its potential effects on physical well-being. A major concern with excessive screen time is its tendency to replace physical activity. As screen use increases, time spent engaging in movement, which is crucial for muscle development, bone health, and energy expenditure, tends to decline. This reduced activity contributes to slower metabolism, loss of muscle mass, and an increased risk of childhood obesity [61]. Screen time is also associated with increased caloric intake. Mindless snacking while watching TV or gaming can lead to overeating, particularly of unhealthy foods, which further contributes to weight gain [62]. Prolonged sitting and reduced movement additionally contribute to metabolic dysregulation, raising risks for conditions such as type 2 diabetes and cardiovascular disease [63].

#### 3.4.1. Effects on Sleep Patterns and Quality

The blue light emitted by screens can suppress melatonin production, a hormone essential for regulating sleep cycles. This disruption can delay sleep onset and lower sleep quality, especially when devices are used close to bedtime [64]. Irregular sleep patterns affect cognitive performance, emotional regulation, and immune function. Children who do not get enough quality sleep may struggle with attention, memory, and mood, and may be at increased risk of developing chronic health issues [65].

#### 3.4.2. Visual Health

Screen overuse is also associated with computer vision syndrome, a condition characterized by eye strain, dry eyes, headaches, and blurred vision after prolonged screen exposure [66]. Moreover, increased screen time—especially up close—is a documented risk factor for myopia (nearsightedness), which is becoming more prevalent in children globally [67].

### 3.5. Language Development

Language development is critical for communication, literacy, and social connection. It includes the ability to comprehend, use vocabulary, and form grammatical structures. With screens now occupying a significant place in children’s daily lives, it is essential to evaluate how screen time influences language learning.

#### 3.5.1. Role of Screen Time in Language Acquisition and Communication Skills

Passive screen time (e.g., TV viewing) offers minimal interactive engagement. Children receive information without opportunities for conversational exchange, which limits essential turn-taking and back-and-forth dialogue that supports language development [68]. Passive viewing can reduce attention span and the ability to engage in language-rich environments [25]. In contrast, interactive screen time (e.g., educational apps) can encourage language learning when designed with development in mind. These platforms can foster vocabulary growth, comprehension, and phonological awareness through responsive and engaging exercises [69].

#### 3.5.2. Comparison Between Passive and Interactive Screen Time

Passive media lacks meaningful interactivity and often fails to support sustained language learning. By contrast, well-designed interactive tools offer engaging, child-directed content that supports verbal expression and cognitive processing. When combined with parental engagement, such platforms can enhance language acquisition and literacy skills [70].

#### 3.5.3. Potential Delays in Language Development Due to Excessive Screen Exposure

A significant consequence of screen overuse is the decline in caregiver–child interaction. Real-time conversation with adults supports the development of syntax, semantics, and social language. Without it, children may lack the scaffolding needed for proper language development [71]. Screen time also limits exposure to diverse language inputs—the rich, complex, and contextually meaningful conversations found in daily interactions. This can result in restricted vocabulary growth and impaired pragmatic language use [72].

Furthermore, children may experience delayed social communication development. Skills such as interpreting body language, managing conversation flow, and understanding tone are best learned in live social settings. Screen time, especially when excessive, may hinder these essential abilities [73]. Excessive use can also impact a child’s attention span, making it harder for them to stay engaged in conversations, storytelling, or group learning, all of which are vital for language development [15].

### 3.6. Neurodevelopmental Effects

With technology becoming more and more ingrained in daily life, research on the effects of screen time on brain development particularly with regard to neuroplasticity and gray matter, has grown significantly. Table 4 summarizes the effect of screen time on neurodevelopment in children [16,17,23,27,42,43].

Numerous studies have examined how screen usage affects kids’ attention and cognitive development, as well as any possible connections to attention problems including attention deficit and hyperactivity disorder (ADHD) and cognitive impairments. Several important conclusions have been drawn from the evidence thus far, even if more research is required to prove clear cause-and-effect relationships:

1. Increased risk of ADHD Symptoms:
  - Attention and focus: Children’s attention spans have been linked to extended screen time, especially when it comes to fast-paced, interactive media (such as social media or video games). Children may find it more difficult to concentrate on less engaging activities (such as homework or chats) as a result [16].
  - Hyperactivity: Excessive screen time, especially when it comprises high-energy content, may be a contributing factor to hyperactive behavior, according to several studies. Although the relationship is complicated and not entirely understood, this is consistent with symptoms of ADHD. While some studies contend that excessive screen time may worsen pre-existing symptoms of ADHD, others imply that children with ADHD may be more drawn to screen-based activities [27].
2. Cognitive delays and developmental concerns:
  - Delayed language development: Language development delays may occur in young children who spend a lot of time on screens. Activities like reading and in-person interactions that are essential for language learning are frequently replaced by screen usage. Verbal communication, vocabulary development, and other cognitive abilities may be impacted by these delays [16].
  - Impact on executive function: Overuse of screens, particularly during early childhood, has been associated with a decline in the development of executive skills like memory, self-control, and problem-solving. Given the importance of executive processes to both academic achievement and general cognitive development, this is especially worrisome [23,42].

### 3.7. Parental Mediation and Screen Time Management

#### 3.7.1. The Role of Parents in Moderating Screen Time Usage

In the current digital era, where screens are a necessary component of everyday life, parents need to actively monitor and establish limits to make sure that technology use stays constructive and in moderation. Table 5 enumerates parental strategies in moderating screen time usage in children [26,74,75].

#### 3.7.2. Mediation Strategies

Techniques that seek to improve or direct kids’ media experiences in a constructive manner are known as positive mediation. Promoting media use and interaction in a healthy way is the main goal. In order to avoid any potential negative impacts, negative mediation techniques usually try to limit or restrict children’s media exposure. These tactics put more of an emphasis on exposure limitation and control. Table 6 enumerates parental mediation strategies in moderating screen time usage in children [18,29,30,31,76].

##### Impact of Parental Screen Time Habits on Children

Given that kids frequently imitate their parents’ behavior, the effects of parental screen time habits on kids are a crucial subject. The quantity and caliber of screen time parents spend with their kids can have a variety of effects on their development.

1. Modeling behavior—Children may learn to spend too much time on screens if their parents do, which could result in them using screens excessively as well [77]. They may develop a dependence on screens if their parents use computers, cell phones, or tablets frequently and subtly teach them that these gadgets are the preferred means of communication or amusement [20].

2. Effects on parent–child interaction—Parents who spend too much time on screens may engage with their kids less in person. Time spent on electronics, particularly social media or work-related activities, has been shown to disrupt genuine, interesting talks between parents and kids [21]. Insufficient in-person engagement can impede emotional attachment, diminishing chances for kids to cultivate empathy, communication abilities, and emotional control. For social and emotional development, children benefit greatly from interaction with their parents [32].

### 3.8. Cultural and Socioeconomic Factors

Children’s screen time and how it is viewed, controlled, and incorporated into daily life are greatly influenced by cultural attitudes toward technology. The degree to which technology is used and how it is perceived in relation to education, entertainment, and socialization are influenced by these views, which differ among nations, communities, and family environments [22,33]. These cultural elements may have the following effects on kids’ screen time:

1. Perceptions of technology’s role in development
  o Positive attitudes: Screen time may be more acceptable in societies that view technology as a necessary educational tool. Increased screen time for activities like learning apps, online learning, and virtual classrooms may be permitted or even encouraged by parents and educators. This is frequently observed in settings that place a high value on the potential of technology to improve education.
  o Negative attitudes: In cultures where there is skepticism about the effects of screen time on child development, particularly its impact on attention span, physical health, and social skills, there may be more restrictions. For instance, some societies place a high value on outdoor play, face-to-face interactions, or hands-on learning, leading to limited use of screens [22].
2. Technological accessibility and affordability
  o Wealthier countries: In more affluent societies where access to personal devices (smartphones, tablets, computers) is widespread, screen time may be higher, with children using technology for entertainment, socializing, and schoolwork. Technology may be integrated into daily life as a necessity, with cultural expectations that children will be adept at using these devices from a young age [33].
  o Developing regions: In lower-income or developing areas, the use of technology may be less common or more restricted. Cultural norms in these areas may prioritize physical activities, community-based education, or traditional play, which can result in less screen time for children. However, access to technology may be increasing, and in some cases, the influence of global trends may shift attitudes.

#### Global Perspectives on Screen Time and Child Development

According to recommendations from the Canadian Paediatric Society (CPS) and the AAP, children under the age of two should not use screens at all, while older children should only use screens for one to two hours per day [22,33]. These recommendations stress that screen time should not get in the way of sleep, exercise, or in-person relationships. Sweden and the UK are two European nations that have adopted progressive approaches to screen time management, with differing degrees of emphasis on children’s digital well-being. Public health programs that emphasize balanced screen usage have also been used in the UK to address this issue [34]. In order to encourage healthier lives, the National Health Service (NHS) advises parents to establish limits and limit their young children’s screen usage [24]. Children are exposed to screens at younger ages in nations like South Korea and Japan, where technology is ingrained in daily life. Nonetheless, South Korea has taken the lead in combating digital addiction by introducing initiatives to assist kids in cutting back on screen time and encouraging better online practices [78]. To curb excessive use, the government of the nation has implemented programs like “Digital Detox” and other measures. China has also put laws into place to safeguard the health of children, especially with regard to video games. Children’s online gaming was restricted by the Chinese government in 2021 to three hours per week [79]. On the other hand, attitudes toward screen time are more varied in nations like India, where excessive screen time is more common in cities, particularly as cell phones get cheaper. On the other hand, screen time is generally lower in rural areas. There is a growing awareness of the need for rules as a result of parents’ increased concerns about screen usage, especially in middle-class urban households.

### 3.9. Potential Benefits of Screen Time

#### 3.9.1. Educational Content and Digital Literacy

When utilized for instructional reasons, screen time can be very advantageous. Access to a wide range of educational resources, including those related to science, math, and languages, is made possible by digital platforms and applications. By encouraging students to participate in interactive learning, these resources can make learning more approachable and enjoyable. Instructional videos, e-books, and educational games may accommodate a variety of learning preferences and make difficult subjects fun for students to understand [35,80].

#### 3.9.2. Development of Technological Skills and Preparedness for Future Careers

Early screen time exposure aids in the development of vital abilities that are essential in today’s workforce. Children and teenagers can learn digital communication, problem-solving techniques, and coding. Well-planned screen time can foster the growth of creativity, critical thinking, and teamwork, particularly when utilizing interactive programs and collaborative platforms [36]. Additionally, since technology has a significant impact on many businesses nowadays, it is becoming more and more crucial for students preparing for future employment to comprehend digital tools, software, and online platforms. Understanding technologies such as cloud computing, artificial intelligence, and virtual reality is crucial for future job preparation because they are revolutionizing many industries [37,44,81]. People can also produce and express themselves artistically through a variety of digital platforms, including graphic design tools, video editing software, and music creation apps. Whether creating video games, podcasts, or digital art, these endeavors encourage creativity and can provide a creative outlet [81].

### 3.10. Interventions and Recommendations

In order to mitigate the detrimental consequences of excessive screen time, a number of interventions and suggestions have been developed as research into these impacts continues. Parental guidance, active involvement, and mediation are some of the best strategies for controlling screen time. The AAP suggests that parents clearly limit their children’s screen use to no more than an hour per day for those between the ages of two and five [38]. Since getting enough sleep is crucial for healthy brain development, cutting back on screen time before bed can enhance the quality of sleep, which supports cognitive processes like memory and emotional control. According to WHO guidelines, children under the age of two should not use screens at all, and those between the ages of two and four should not spend more than an hour a day on them [82]. Encouraging alternative activities like social engagement, outdoor play, and physical activity balances out the sedentary behavior that is frequently linked to excessive screen usage. Establishing areas of the house where screens are not allowed can be a successful intervention [39,40]. Not all screen time is bad, particularly if kids are interacting with instructional materials that encourage active learning. Interactive games and age-appropriate programming have been shown to enhance cognitive abilities like memory, problem-solving, and language development. Prioritizing interactive media above passive content is crucial. Particularly helpful for cognitive growth are apps and games that promote critical thinking, problem solving, or teamwork [41,83]. Additionally, communities and schools may make a significant contribution by putting in place educational initiatives that emphasize the value of consuming media in moderation. Together, families, schools, and medical professionals may establish a nurturing atmosphere that promotes responsible media use [38,82].

#### Study Limitations

The studies that were reviewed had certain limitations. The consistency of results was impacted by variations in study design, sample size, and outcome measurement instruments. Additionally, a number of studies failed to take into consideration potential confounders that could affect the results, such as parental influence, socioeconomic position, and pre-existing medical issues.

## 4. Conclusions

Children’s development is impacted by screen time in a variety of ways, both positively and negatively. The hazards linked with excessive screen time can be reduced by implementing treatments like parental mediation, screen exposure limits, non-screen activities, and the promotion of high-quality educational content. Clear policy guidelines and public health measures are also necessary to help families effectively manage screen use. Children will be able to navigate the digital age in a way that promotes healthy cognitive, emotional, and social development with the support of ongoing research, cooperation, and education.

## Figures and Tables

**Table 1:** Study characteristics and key findings.

Author and Year [Reference Number]	Country	Study Design	Screen Type	Sample Size	Key Findings
Fardouly, 2015 [12]	Australia, UK	RCT	Facebook	112 females	Participants who spent time on Facebook reported being in a more negative mood than those who spent time on the control (fashion magazine; neutral control website)
Nesi, 2015 [13]	USA	Longitudinal study	Cell phones, Facebook, and Instagram	619; (57.3% females) ages of 12 and 16 (mean age 14.6)	Short Mood and Feelings Questionnaire was used to assess depressive symptoms. Significant positive associations were found between the frequency of technology use, technology-based SCFS, and offline excessive reassurance-seeking behaviors. Popularity was positively associated with frequency of technology use and technology-based SCFS; however, it was negatively associated with depressive symptoms.
Coyne, 2020 [14]	USA	Longitudinal study	Social media (Facebook, Instagram)	500; 10 and 13 years (51.6% female)	Self-reported Center for Epidemiological Studies Depression Scale for Children, and generalized anxiety disorder subscale from the Spence Child Anxiety Inventory were used. Time spent using SNSs were moderately related to anxiety and depression.
Christakis, 2018 [15]	USA	Longitudinal study	Television	1278 children at age 1 and 1345 children at age 3	Increased television viewing before the age of 3 was associated with increased risk of attentional problems at 7 years of age. The pacing of shows drove these effects, with faster pacing having stronger associations with subsequent attentional problems.
Hutton, 2020 [16]	USA	Cross-sectional study	Screen time	47; aged 3 to 5 years (54.3 ± 7.5 months)	In healthy prekindergarten children, screen use greater than that recommended was associated with (1) lower measures of microstructural organization and myelination of brain white matter tracts that support language and emergent literacy skills and (2) corresponding cognitive assessments.
Horowitz-Kraus, 2015 [17]	USA	Comparative study	Screen-based media time (smartphones/tablets/desktop/laptop computers/television)	19; aged 8–12 years	MRI assessed resting-state connectivity between the left visual word form area, as the seed area, and other brain regions. Time spent reading was positively correlated with higher functional connectivity between the seed area and left-sided language, visual, and cognitive control regions. Screen time was related to lower connectivity between the seed area and regions related to language and cognitive control.
McArthur, 2021 [3]	Canada	Cohort	TV, computer, video games	1994 mothers and children; 36 months	Duration of screen time is associated with poor child development outcomes. Compared to ≤1 h/day, children using screens 2 h or ≥3 h/day had an increased likelihood of reported behavioral problems (adjusted odds ratio (AOR) 1.30–1.90), delayed achievement of developmental milestones (AOR 1.41–1.68), and poorer vocabulary acquisition (AOR 1.94).
Chng, 2015 [18]	Singapore	Interventional study	Internet use	3079	Restrictive mediation was found to be negatively associated with pathological Internet use. This relation was stronger for higher levels of attachment, communication, and comfort at home, implying that the effectiveness of restrictive mediation varies with the degree of warmth and support in the general family environment.
Lillard, 2015 [19]	USA	Observational 3 studies	Shows on screen; fast fantastical shows vs. slow, realistic shows	300; aged 4–6 years	Executive function is impaired after watching fast and fantastical shows, relative to that of children who watched a slow, realistic show or played. Only fantastical shows, regardless of their pacing, disrupted 4-year-olds’ executive function.
Chou, 2017 [20]	Taiwan	Cross-sectional survey	Internet use	3169; aged d13–16	Quality of Internet parenting behaviors may be related to changes in students’ internet expectancy and addiction. Children’s amount of Internet use, their gender, and parental education level are additional factors.
Collier, 2016 [21]	USA	Meta-analyses	Effect of parental mediation of media on child outcomes (aggression, sexual behaviors)	-	Small but significant relationships between child outcomes and restrictive mediation (r+ = −0.06), and co-viewing (r+ = 0.09). Active mediation was individually related to lower levels of aggression (r+ = −0.08), sexual behavior (r+ = −0.06), and substance use (r+ = −0.11)
Fardouly, 2018 [4]	Australia	Observational study	Parental control over the time their child spends on social media	N = 284; aged 10–12	Preadolescents, whose parents reported greater control over their child’s time on social media, reported better mental health. This relationship was mediated by preadolescents spending less time browsing and making fewer appearance comparisons on social media.
Canadian Paediatric Society, 2019 [22]	Canada	Guidance	Promoting health and development	Children less than 5 years of age	Co-viewing quality screen content can positively influence children’s social adaptive skills, sleep patterns, and behaviors by being involved with and setting limits on their screen time. TV viewing negatively associated with school readiness skills.
American Academy of Pediatrics, 2016, 2022 [23]	USA	Review	Television, videos, and mobile/interactive technologies	Children less than 5 years of age	Heavy media use during preschool years is associated with small but significant increases in BMI; fewer minutes of sleep per night; and cognitive, language, and social/emotional delays. Excessive exposure to screens (television, tablets, smartphones, computers, and video game consoles), especially at young ages, is associated with lower academic performance, sleep disturbances, obesity, attention deficit, increased aggression, lower self-esteem, depression, and increased rates of high-risk behaviors.
National Health Service guidance, 2023 [24]	UK	Advice	Television, video games, electronic devices	School-age children	Recommended no more than 2 h of screen time for good sleep and the prevention of obesity, alongside other measures.
Bedford, 2016 [25]	UK	Observational study	Touchscreen	366; aged 19–36 months	In toddlers, aged 19–36 months, age of first touchscreen use was significantly associated with fine motor skills (stacking blocks) (*p* = 0.03) after controlling for covariates age, sex, mother’s education (a proxy for socioeconomic status) as well as age of early fine motor milestone achievement (pincer grip). This effect was only present for active scrolling of the touchscreen (*p* = 0.04), not for video watching. No significant relationships were found between touchscreen use and either gross motor or language milestones.
Anderson, 2017 [26]	USA	Review	Screen and media	Age group: toddlers to adolescents	For children <2 years old, television viewing has mostly negative associations, especially for language and executive function. For preschool-aged children, television viewing has been found to have both positive and negative outcomes, and a large body of research suggests that educational television has a positive impact on cognitive development.
Madigan, 2019 [27]	Canada	Longitudinal cohort study	Screen time	2441; 24, 36, and 60 months	Higher levels of screen time at 24 and 36 months were significantly associated with poorer performance on developmental screening tests at 36 months (β, −0.06; 95% CI, −0.10 to −0.01) and 60 months (β, −0.08; 95% CI, −0.13 to −0.02), respectively. These within-person (time-varying) associations were statistically controlled for between-person (stable) differences.
Twenge, 2019 [28]	USA	Observational study	Screen time	8.2 million; aged 13–18 years	Adolescents low in in-person social interaction and high in social media use reported the most loneliness.
Paulus, 2019 [29]	USA	Cohort	Screen media activity (SMA)	4524; adolescents	Some SMA-related factors corresponded with higher externalizing (Cohen’s d effect size (ES) 0.06–0.1) but not internalizing psychopathology and lower crystalized (ES: 0.08–0.1) and fluid intelligence (ES: 0.04–0.09). Findings support the notion of SMA-related maturational coupling or structural correlation networks in the brain and provide evidence that individual differences of these networks have mixed consequences for psychopathology and cognitive performance.
Crone, 2018 [30]	Netherland	Review	Media use	-	Neural systems that are associated with behaviors that are important for social media use, including social reward processing, emotion-based processing, regulation, and mentalizing about others. As these neural systems are still underdeveloped and undergoing significant changes during adolescence, they may contribute to sensitivity to online rejection, acceptance, peer influence, and emotion-loaded interactions in media environments.
Carter, 2016 [31]	UK	Review	Media device use	125 198; mean [SD] age, 14.5 [2.2] years	There was a strong and consistent association between bedtime media device use and inadequate sleep quantity (odds ratio [OR], 2.17; 95% CI, 1.42–3.32) (*p* < 0.001, I2 = 90%), poor sleep quality (OR, 1.46; 95% CI, 1.14–1.88) (*p* = 0.003, I2 = 76%), and excessive daytime sleepiness (OR, 2.72; 95% CI, 1.32–5.61) (*p* = 0.007, I2 = 50%).
Cheung, 2017 [32]	UK	Observational study	Television and touchscreens	715; 6–36 months	Increased TV exposure associated with less sleep during the day. Increased touchscreen use was associated with decreased overall amount of sleep (beta = −0.146, SE = 0.049, *p* = 0.003). The unstandardized beta value (−0.26) means that every additional hour of touchscreen use is associated with an overall reduction in sleep of 15.6 minutes.
Twenge, 2017 [33]	USA	Observational study	Electronic device use, social media, and reading online	369,595; adolescents	Clear exposure–response relationship of short sleep duration with the use of electronic devices after 2 or more hours per day
Vernon, 2018 [34]	Australia	Observational study	Mobile phone	1101; aged 13–16 years	Increased night-time mobile phone use was directly associated with increased externalizing behavior and decreased self-esteem and coping. Changes in sleep behavior increase depressed mood and externalizing behavior, and later declines in self-esteem and coping.
Grover, 2016 [35]	USA	Observational study	Mobile phone	2353; adolescents	Longer duration of messaging after lights out was more likely to lead to a shorter sleep duration, a higher rate of daytime sleepiness, and poorer academic performance.
Twenge, 2018 [36]	USA	Observational study	Cell phones, computers, electronic devices, games, and TV	40,337; aged 2–17 years	After 1 h/day of use, more hours of daily screen time were associated with lower psychological well-being, including less curiosity, lower self-control, more distractibility, more difficulty making friends, less emotional stability, being more difficult to care for, and inability to finish tasks. Among 14- to 17-year-olds, high users of screens (7+ h/day vs. low users of 1 h/day) were more than twice as likely to ever have been diagnosed with depression (RR 2.39, 95% CI 1.54, 3.70), ever diagnosed with anxiety (RR 2.26, CI 1.59, 3.22), treated by a mental health professional (RR 2.22, CI 1.62, 3.03) or have taken medication for a psychological or behavioral issue (RR 2.99, CI 1.94, 4.62) in the last 12 months. Moderate use of screens (4 h/day) was also associated with lower psychological well-being
Grontved, 2015 [37]	Denmark	Longitudinal cohort	Television, computer, total screen time	435; up to 12 years	Each additional hour/day spent screen viewing in adolescence was associated with 1.36 (95% CI 0.73–1.98) and 1.05 (95% CI 0.50–1.60), respectively, greater depression summary score in young adulthood (*p* < 0.001), and 1.64 (95% CI 1.18–2.27) and 1.58 (95% CI 1.18–2.12), respectively, greater odds of prevalent depression in young adulthood, and dose–response relationships were indicated.
Maras, 2015 [38]	Canada	Observational Study	Television, video games, and computers	2482; Grade 7 to 12 students	Duration of screen time was associated with severity of depression (β = 0.23, *p* < 0.001) and anxiety (β = 0.07, *p* < 0.01). Video game playing (β = 0.13, *p* < 0.001) and computer use (β = 0.17, *p* < 0.001) but not TV viewing were associated with more severe depressive symptoms. Video game playing (β = 0.11, *p* < 0.001) was associated with the severity of anxiety.
Liu, 2015 [39]	China	Systematic review	Total screen time	127,714	Higher screen time (ST) in preadolescent children and adolescents was significantly associated with a higher risk of depression (OR = 1.12; 95% CI 1.03 to 1.22). Screen type, age, population and reference category acted as significant moderators. Compared with those with no ST, there was a non-linear dose–response association of ST with a decreasing risk of depression at ST < 2 h/day, with the lowest risk being observed for 1 h/day (OR = 0.88; 95% CI 0.84 to 0.93).
Twenge, 2018 [40]	USA	Observational study	Screen time	1.1 million; 8th, 10th, and 12th graders	Adolescents who spent more time on electronic communication and screens (e.g., social media, the Internet, texting, gaming) and less time on non-screen activities had lower psychological well-being.
Parent, 2016 [41]	USA	Observational study	Television, computers, smartphones, video games, tablets	3–7 yrs; [N = 209], 8–12 yrs; [N = 202], 13–17 yrs; [N = 210]	Higher levels of youth screen time were associated with more sleep disturbances, which, in turn, were linked to higher levels of youth behavioral health problems.
Radesky, 2016 [42]	USA	Review	Mobile and interactive media	0–8 years	Excessive media use negatively affects child cognitive, language, literacy, and social–emotional development.
Madigan, 2020 [43]	Canada	Systematic review and meta-analysis	Total screen time	18,905; aged up to 12 years	According to data from 42 studies, greater quantity of screen use (i.e., hours per day/week) was negatively associated with child language, while better quality of screen use (i.e., educational programs and co-viewing with caregivers) was positively associated with child language skills (screen time [n = 38; r = −0.14; 95% CI, −0.18 to −0.10]; background television [n = 5; r = −0.19; 95% CI, −0.33 to −0.05]), while better-quality screen use (educational programs [n = 13; r = 0.13; 95% CI, 0.02–0.24]; co-viewing [n = 12; r = 0.16; 95% CI, 0.07–.24]) were associated with stronger child language skills. Later age at screen use onset was also associated with stronger child language skills [n = 4; r = 0.17; 95% CI, 0.07–0.27].

**Table 2:** AAP guidelines for screen time usage.

Age Group	Recommendation	Rationale
Children under 18 months	Avoid screen time, except for video chatting.	Sensory-rich and interactive play promotes brain development more effectively than screens [2].
Children 18 to 24 months	Introduce high-quality digital content only with caregiver co-viewing	Adult interaction enhances learning and understanding [2].
Children 2 to 5 years	Limit screen use to one hour daily of high-quality content, with active adult involvement	Encourages better comprehension and mitigates passive use [2].
Children 6 years and older	Create consistent screen time limits; prioritize physical activity, sleep, and interpersonal interaction	Helps develop healthy life balance and habits [2].
Teens	Allow screen use for education and social connection but set clear limits on entertainment and bedtime usage	Promotes sleep hygiene and family engagement [3].

**Table 3:** Effect of screen time on social and emotional development.

Domains	Effect on Social and Emotional Development
Influence on social skills and relationships	Excessive screen time often reduces face-to-face interaction, which is vital for the development of core social skills like turn-taking, active listening, and reading nonverbal cues. These interpersonal abilities are typically acquired through real-life social interactions [51]. Impaired nonverbal communication. Children need to engage in conversations where they can learn to interpret facial expressions, tone of voice, and body language. Prolonged screen time, particularly passive media use, can impede this learning process, making it harder for children to decode social signals and respond appropriately in face-to-face interactions [52]. Development of empathy. Empathy and compassion grow through direct social experiences that involve understanding others’ perspectives and emotions. When screen-based activities take the place of in-person interactions, children may miss important opportunities to develop these emotional skills [53]. Peer relationships may also suffer. Children who spend more time with screens than with peers can become socially isolated. They may experience difficulties initiating and maintaining friendships, participating in group settings, or resolving interpersonal conflicts peacefully [28].
Effects on emotional regulation and behavioral issues	Screen exposure, especially to violent or aggressive content, can lead to behavioral changes, including increased aggression. According to the American Psychological Association, media violence exposure may desensitize children to violence, reinforcing aggressive behavior patterns [54]. Anxiety and depression are increasingly associated with excessive screen use. Teenagers are particularly vulnerable due to social comparison, fear of missing out (FOMO), and the relentless flow of digital stimuli that can lead to poor sleep and low self-esteem [55]. Attention difficulties—Fast-paced screen content may impair a child’s ability to concentrate on slower-paced and real-world tasks, contributing to both emotional dysregulation and behavioral issues [56]. Some children may also develop a dependency on screens for emotional self-soothing, turning to them for distraction rather than learning healthy coping mechanisms such as mindfulness, communication, or physical activity [57].
Impact of excessive screen time on self-esteem and body image	Children and teens are highly impressionable, and idealized images on social media can set unrealistic beauty standards. Constant exposure to these polished portrayals can fuel body dissatisfaction and lower self-esteem, especially in adolescents [12]. Social comparison is another harmful consequence. Viewing others’ curated lifestyles on social media platforms can lead children to feel inadequate, fostering internalized pressure to meet unrealistic expectations [13]. Cyberbullying has become a prevalent issue, particularly due to the anonymity and reach of digital platforms. Victims of cyberbullying often experience intense emotional distress, depression, and significant damage to their self-worth [58].
Social isolation and screen time’s role in social skills development	Excessive screen use can lead to reduced social engagement, limiting children’s opportunities to practice essential interpersonal skills in real-world settings. While digital communication can maintain connections, it often lacks the depth and nuance of in-person interactions [59]. Children who rely heavily on screens may struggle with initiating conversations, resolving disputes, or participating in group activities, competencies that are best developed through face-to-face experiences [14]. Although online interactions can serve a social function, research suggests that they rarely substitute for the emotional and psychological richness of offline relationships, which are more effective for nurturing trust, empathy, and social confidence [60].

**Table 4:** The effect of screen time on neurodevelopment.

Domains	Description	Potential Positive Effects	Potential Negative Effects
Neuroplasticity and screen time	The brain’s capacity to rearrange itself by creating new synaptic connections throughout life, particularly in reaction to experience and learning, is known as neuroplasticity. Numerous factors, including environmental stressors like screen time, influence the nature of these alterations.	Cognitive training: By involving attention, memory, and decision-making processes, some screen activities (such as instructional apps and problem-solving video games) may encourage neuroplasticity. This can aid in the development of cognitive abilities, especially when screen time is instructive and participatory [43]. Memory and learning: Interactive digital platforms can promote the formation of new neural connections in parts of the brain linked to memory and learning, such as the hippocampus. Digital tools that demand multi-tasking or strategy-based games may activate distinct brain regions, which could improve cognitive flexibility, spatial awareness, and memory recall [16,17].	Reduced attention span: When compared to activities that demand active thinking, passive screen time, such as watching TV or browsing social media, may cause the brain to engage less. The brain may grow more used to a low-stimulation, less demanding environment as a result of this passive intake, which could decrease neuroplasticity in regions linked to learning and attention [27]. Impaired development of executive functions: Overuse of screens, especially by young children, can interfere with the development of executive functions (such as working memory and impulse control) by reducing participation in interactive, real-world, and physically demanding activities that foster these abilities [42].
Gray matter and screen time	Neuronal cell bodies make up gray matter, which is essential for information processing and cognitive processes like decision making, muscle control, and sensory perception. Changes in gray matter density or volume may be a reflection of changes in how the brain develops and functions.	Cognitive and emotional development: Some gray matter regions, especially those linked to learning and problem-solving, may benefit from moderate screen use for educational purposes. For example, playing video games that demand strategy, logic, and the ability to learn from errors may promote the formation of gray matter in regions related to cognitive flexibility and decision-making [43]. Memory and learning: Research indicates that using digital learning resources could improve gray matter in memory-related areas (such as the hippocampus). When the screen content is intended to be both instructional and cognitively challenging, this effect is particularly apparent [17].	Reduced gray matter in children and adolescents: According to research, too much screen time, especially when it takes the place of exercise or sleep, may cause the prefrontal cortex and other areas linked to executive functioning and sensory processing to lose gray matter density [17]. Long-term effects on cognitive functions including memory and attention may result from this. Impact on social and emotional development: Overuse of screens can reduce in-person social interactions, which are essential for the growth of social cognition and emotional control. Gray matter reductions in areas of the brain implicated in these processes, such as the prefrontal cortex and amygdala, have been connected to a lack of real-world socialization [23].

**Table 5:** Parental strategies in moderating screen time usage.

Strategy	Description
1. Setting clear boundaries and guidelines	Given the child’s age, needs, and activities, parents should establish clear and sensible screen time limitations. While older children may need limitations that balance screen time with other important activities like homework, housework, and physical activity, the AAP recommends that children aged 2 to 5 should not spend more than an hour a day on screens [26].
2. Modeling healthy screen habits	Kids pick up a lot of knowledge from watching their parents. Good screen habits, such as setting screen time limits and emphasizing in-person interactions, should be modeled by parents. Children are more inclined to follow their parents’ example of responsible technology use [74].
3. Promoting physical and social activities	Social contacts, outdoor play, and physical activity should all be promoted by parents. In addition to supporting the child’s general development, both socially and physically, these activities can help balance screen usage. Children’s excessive screen time can be avoided by promoting pastimes like reading, athletics, or artistic endeavors like music or sketching [75].

**Table 6:** Mediation strategies.

Positive mediation strategies	Co-viewing (watching together): Children watch media with their parents or other caregivers. This method enables cooperative attention, participation, and the chance for adults to set the scene, explain details, and resolve any issues. Co-viewing allows parents to set an example of proper media conduct or attitudes while also assisting youngsters in processing the content, e.g., watching a teaching program together and then talking about the facts or ideas afterwards [76]. Active mediation (discussion and guidance): Actively discussing, clarifying, probing, and promoting critical thinking with kids about the material they are consuming is beneficial. Children are better able to comprehend the material, consider the contents, and pose questions as a result. It can teach kids media literacy and encourage a deeper interaction with media, e.g., asking a child to consider the meaning of an advertisement or talking about the moral lessons of a show [29]. Encouraging educational content: Children can be directed toward age-appropriate, instructive, or enriching information by their parents. This method encourages learning while advancing emotional and cognitive growth. Children’s social skills, language development, and problem-solving abilities can all be improved via educational media, e.g., encouraging kids to watch instructional cartoons, documentaries, or learning-focused applications [30].
Negative mediation strategies	Limiting screen time: Parents frequently set stringent limits on how much time their kids spend on screens, such as no more than an hour each day. This strategy aids in avoiding overexposure to media, which has been connected to detrimental effects like decreased physical activity, disturbed sleep, and a lack of social interaction, e.g., establishing a timer that, after a predetermined period of time, automatically shuts off the TV or app [31]. Content restriction (blocking or filtering): In order to prevent unsuitable content or restrict screen time to specific apps, parents frequently use software tools or device settings to restrict access to particular sorts of content. It helps shield kids from improper or dangerous content. Additionally, it might keep kids away from excessive or addictive information, e.g., blocking websites with violent or pornographic material with parental control software [18].

## Data Availability

No new data were created or analyzed in this study. Data sharing is not applicable to this article.
